# Electrochemical gradients are involved in regulating cytoskeletal patterns during epithelial morphogenesis in the *Drosophila* ovary

**DOI:** 10.1186/s12861-019-0203-y

**Published:** 2019-11-12

**Authors:** Isabel Weiß, Johannes Bohrmann

**Affiliations:** Institut für Biologie II, Abt. Zoologie und Humanbiologie, RWTH Aachen University, Worringerweg 3, 52056 Aachen, Germany

**Keywords:** *Drosophila melanogaster*, Bioelectricity, Intracellular pH, Membrane potential, Cell polarity, Pattern formation, Ion pump, Ion channel, Microfilament, Microtubule

## Abstract

**Background:**

During *Drosophila* oogenesis, the follicular epithelium differentiates into several morphologically distinct follicle-cell populations. Characteristic bioelectrical properties make this tissue a suitable model system for studying connections between electrochemical signals and the organisation of the cytoskeleton. Recently, we have described stage-specific transcellular antero-posterior and dorso-ventral gradients of intracellular pH (pH_i_) and membrane potential (V_mem_) depending on the asymmetrical distribution and/or activity of various ion-transport mechanisms. In the present study, we analysed the patterns of basal microfilaments (bMF) and microtubules (MT) in relation to electrochemical signals.

**Results:**

The bMF- and MT-patterns in developmental stages 8 to 12 were visualised using labelled phalloidin and an antibody against acetylated α-tubulin as well as follicle-cell specific expression of GFP-actin and GFP-α-tubulin. Obviously, stage-specific changes of the pH_i_- and V_mem_-gradients correlate with modifications of the bMF- and MT-organisation. In order to test whether cytoskeletal modifications depend directly on bioelectrical changes, we used inhibitors of ion-transport mechanisms that have previously been shown to modify pH_i_ and V_mem_ as well as the respective gradients. We inhibited, in stage 10b, Na^+^/H^+^-exchangers and Na^+^-channels with amiloride, V-ATPases with bafilomycin, ATP-sensitive K^+^-channels with glibenclamide, voltage-dependent L-type Ca^2+^-channels with verapamil, Cl^−^-channels with 9-anthroic acid and Na^+^/K^+^/2Cl^−^-cotransporters with furosemide, respectively. The correlations between pH_i_, V_mem_, bMF and MT observed in different follicle-cell types are in line with the correlations resulting from the inhibition experiments. While relative alkalisation and/or hyperpolarisation stabilised the parallel transversal alignment of bMF, acidification led to increasing disorder and to condensations of bMF. On the other hand, relative acidification as well as hyperpolarisation stabilised the longitudinal orientation of MT, whereas alkalisation led to loss of this arrangement and to partial disintegration of MT.

**Conclusions:**

We conclude that the pH_i_- and V_mem_-changes induced by inhibitors of ion-transport mechanisms simulate bioelectrical changes occurring naturally and leading to the cytoskeletal changes observed during differentiation of the follicle-cell epithelium. Therefore, gradual modifications of electrochemical signals can serve as physiological means to regulate cell and tissue architecture by modifying cytoskeletal patterns.

## Background

Localised ion fluxes, gradients of ion concentrations, of intracellular pH (pH_i_) and of membrane potentials (V_mem_) are involved, as fast and wide-ranging signals, in various developmental and regenerative processes [1–6]. Gradual changes of bioelectrical properties mediate diverse cellular events, e.g. proliferation [7], migration [8] and differentiation [9–12]. Establishing electrochemical gradients within single cells or whole tissues requires asymmetrically distributed or activated ion-transport mechanisms [13–16] as well as gap junctions [17–21].

Electrochemical signals are transduced, perceived and translated into cellular responses by pH_i_- or V_mem_-sensitive ion-channels, phosphatases, transporters of signalling molecules or other proteins, like elements of the cytoskeleton [2, 22]. Influences of pH_i_ on actin self-assembly [23], on contractility of the actomyosin cytoskeleton [24] and on the activity of cross-linking proteins [25, 26] are known to exist. Furthermore, changes of V_mem_ are associated with reorganisation or stabilisation of the microfilament (MF) network [27, 28]. Besides pH_i_-dependence of polymerisation as well as depolymerisation of microtubules (MT) [25, 29, 30], correlations between V_mem_-changes and an altered MT-organisation are also known [31–33].

**Fig. 1:**
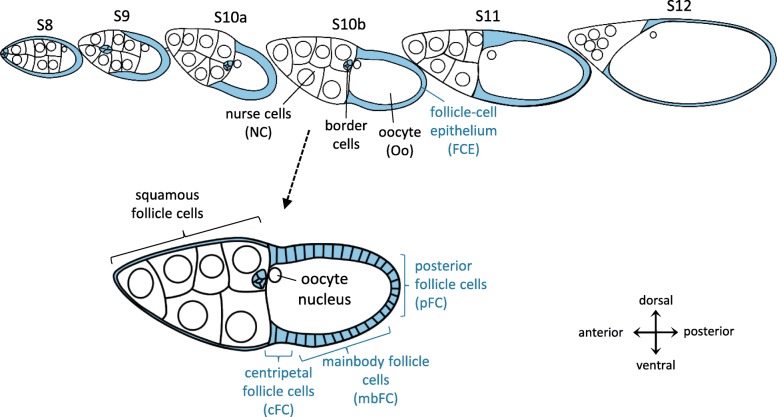
Schematic drawing of the analysed stages of oogenesis. The somatic follicle-cell epithelium (FCE) that surrounds the 15 nurse cells (NC, anterior) and the oocyte (Oo, posterior) is highlighted in blue. During vitellogenic stages 8–12 (S8–12), the FCE undergoes morphological changes and differentiates into several distinct follicle-cell (FC) populations: squamous FC, surrounding the NC, border cells, centripetally migrating FC (cFC), mainbody FC (mbFC) and posterior FC (pFC), surrounding the Oo. From S10b onward, the dorsal FCE (defined by the position of the Oo nucleus) becomes thicker than the ventral FCE. Now, the Oo constitutes almost one half of the follicle’s volume

We have found, in ovarian follicles of *Drosophila melanogaster,* stage-specific patterns of extracellular currents [34], gradients of pH_i_ [15, 16] and gradients of V_mem_ [15, 16, 35]. It is tempting to assume that these bioelectrical phenomena, resulting mainly from the exchange of protons, potassium ions and sodium ions [35–39], serve as signals to guide development. During the course of oogenesis, follicles consisting of 16 germ-line cells, i.e. 15 nurse cells (NC) and one oocyte (Oo), surrounded by a single-layered somatic follicle-cell epithelium (FCE) are passing through 14 stages (S1–14) [40] (Fig. 1). The FCE differentiates into several morphologically distinct follicle-cell (FC) populations [41–43] with characteristic cytoskeletal patterns. Therefore, the FCE is an appropriate model system for studying influences of bioelectrical signals on the cytoskeletal organisation during development. The FCE participates in establishing the embryonic axes [44–46] and in synthesising the multi-layered eggshell [43]. Polarised and parallel aligned MF-bundles (bMF) at the basal side of the FCE have long been assumed to be involved, as a molecular corset, in shaping the egg [47, 48]. Recent studies have demonstrated the role of bMF, and also of MT, during follicle elongation, a complex process which includes a global rotation of the FCE during S5–8 [49–53].

**Fig. 2:**
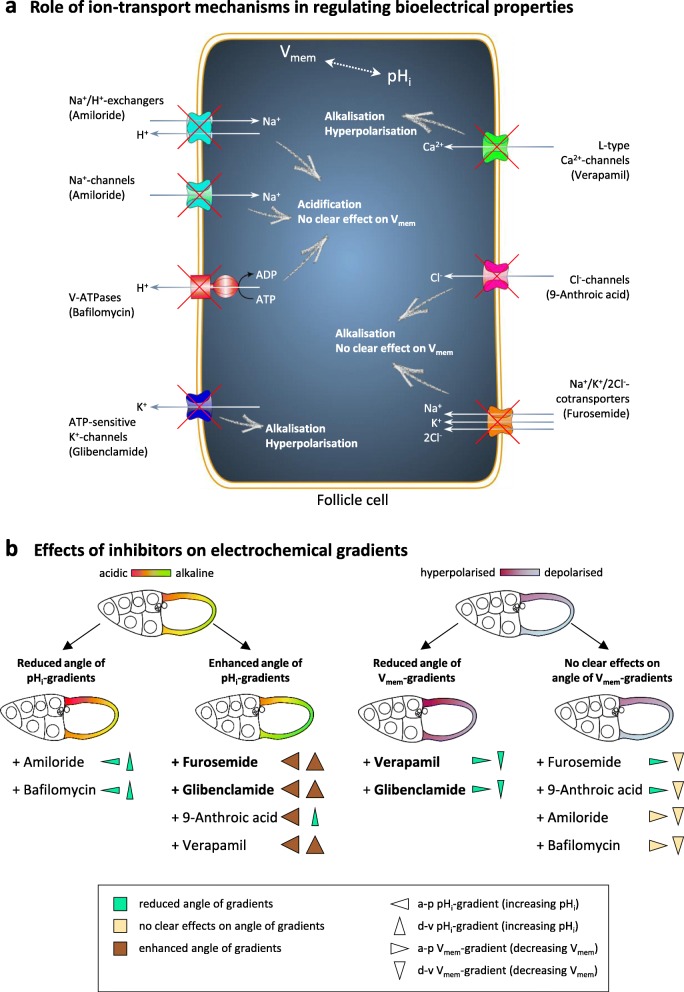
Bioelectrical properties were modified using inhibitors of ion-transport mechanisms (summarised according to [16]). **a** Schematic drawing of a follicle cell showing the analysed ion-transport mechanisms. Na^+^/H^+^-exchangers (NHE) and Na^+^-channels were blocked with amiloride, V-ATPases with bafilomycin, ATP-sensitive K^+^-channels with glibenclamide, voltage-dependent L-type Ca^2+^-channels with verapamil, Cl^−^-channels with 9-anthroic acid and Na^+^/K^+^/2Cl^−^-cotransporters with furosemide. Intracellular pH (pH_i_) and membrane potential (V_mem_) were analysed in living follicles using the pH-indicator 5-CFDA,AM (5-carboxyfluorescein diacetate, acetoxymethyl ester) and the potentiometric dye DiBAC_4_(3) (bis-(1,3-dibutylbarbituric acid) trimethine oxonol). pH_i_, V_mem_ or both parameters were affected by each inhibitor [16]. **b** Schematic summary of the effects of inhibitors on the electrochemical gradients in the columnar FCE during S10b [16]. The antero-posterior (a-p) and dorso-ventral (d-v) pH_i_- and V_mem_-gradients are visualised as colour gradients in the FCE. Triangles symbolise directions of the gradients. Increasing pH_i_ means more alkaline, decreasing V_mem_ means less hyperpolarised. The effects of inhibitors on the angles of the gradients are represented by width and colour of the triangles. While the strongest effects on pH_i_-gradients were generated by furosemide or glibenclamide, the strongest effects on V_mem_-gradients were generated by verapamil or glibenclamide (bold letters) [16]

The aim of the present study is to characterise the physiological relevance of electrochemical gradients by investigating their influence on the cytoskeletal organisation during *Drosophila* oogenesis. We observed stage-specific bMF- and MT-patterns in the FCE and found correlations with the stage-specific bioelectrical patterns described previously [16]. In addition, we used inhibitors of various ion-transport mechanisms, which we have recently shown to modify pH_i_ and V_mem_ as well as the respective gradients during S10b (Fig. 2; [16]). We detected alterations of the bMF- and MT-patterns that result from changes in pH_i_- and V_mem_-gradients and discuss the potential mechanisms.

## Results

### Stage-specific patterns of basal microfilaments

**Fig. 3:**
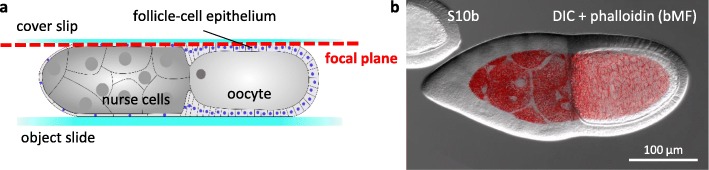
Optical sectioning using structured-illumination microscopy. **a** Schematic drawing of a S10b-follicle placed between an object slide and a cover slip. The focal plane of tangential optical sections to analyse basal microfilaments (bMF) and microtubules (MT) in the FCE is shown as dashed red line. **b** Overlay of a differential interference-contrast (DIC) image and a fluorescent-phalloidin image showing the analysed area of bMF in the FCE (and, in addition, in the NC) in S10b

**Fig. 4:**
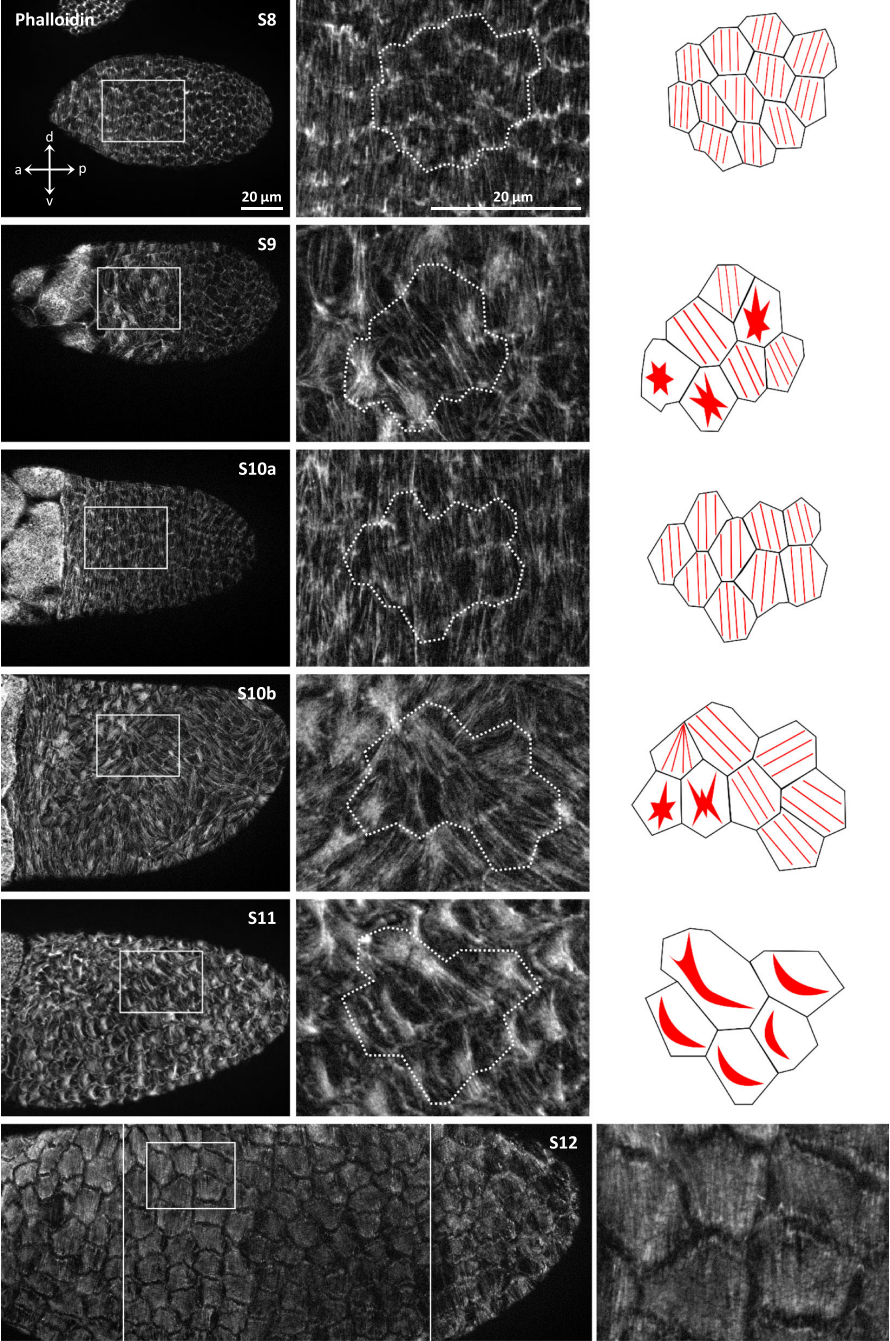
Development of the organisation of basal microfilaments (bMF) in the FCE during S8–12. Tangential optical sections (see Fig. 3) of typical follicles stained with fluorescent phalloidin are shown. Stage-specific features of the bMF-pattern are represented as sketches in the right column. The dotted lines in the middle column (magnifications of boxed areas in the left column) correspond to the lateral FC-membranes seen at a deeper focal plane. Due to cell protrusions close to the basement membrane, the cell borders appear to be shifted. In the cuboidal FC of S8, the preferential bMF-bundle orientation within individual FC as well as relative to neighbouring FC is perpendicular to the follicle’s antero-posterior (a-p) axis. During S9–11, rearrangements of the bMF-organisation occur. In S9, the bMF in the flattening cFC condense (red asterisks), whereas in S10a, the bMF-bundles in cFC are again aligned in parallel perpendicular to the a-p axis, i. e. along the dorso-ventral (d-v) axis. In S10b, condensation followed by disintegration of bMF become obvious in dorsal cFC as well as neighbouring FC, and this pattern spreads out toward the pFC in S11 (crescent-shaped condensations). During S12, a new pattern of dense parallel bMF perpendicular to the a-p axis emerges. In contrast to earlier stages, FC borders are discernible in this focal plane due to chorion ridges. For abbreviations, see legend to Fig. 1. Scale bars refer to all pictures in the same column

We analysed in detail, during vitellogenic stages S8–12, the bMF-organisation in the cuboidal and columnar FCE (Fig. 3) and detected, despite of some variation, characteristic stage-specific patterns (Fig. 4). The bMF-bundles in the cuboidal FCE of S8 are highly polarised perpendicular to the antero-posterior (a-p) axis of the follicle (circumferential organisation). This parallel alignment, both within individual FC and in relation to neighbouring FC, disappears in part during S9. In the flattening FC (the prospective cFC) near the border between NC and Oo, condensations of bMF become obvious. The bMF-bundles in the remaining columnar FCE surrounding the Oo retain their parallel alignment within individual FC, but they become more disordered relative to neighbouring FC. During S10a, the bMF-bundles in cFC are again aligned in parallel and oriented circumferentially. Subsequent morphological changes during S10b, like thickening of the dorsal FCE and elongation of inwardly migrating cFC, are accompanied by bMF-condensations that first appear in dorsal cFC and spread out over mbFC to pFC during S11. In S11, a peculiar bMF-organisation showing crescent- or fan-shaped condensations becomes obvious, wheras during S12, a new pattern of dense parallel bMF oriented circumferentially appears (Fig. 4).

### Stage-specific patterns of microtubules

**Fig. 5:**
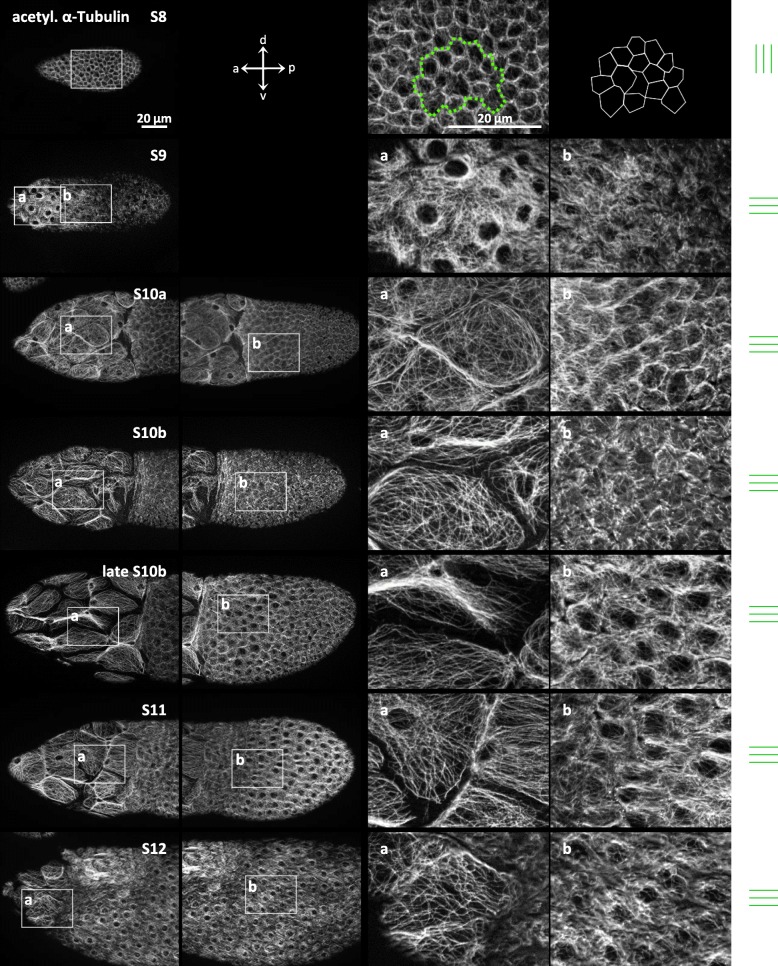
Development of the organisation of microtubules (MT) in the FCE during S8–12. Tangential optical sections (see Fig. 3) of typical anti-acetylated α-tubulin-treated follicles are shown. In the cuboidal FC of S8, the preferential MT-orientation is, similar to the bMF-orientation, perpendicular to the follicle’s a-p axis (indicated as parallel green lines at the right margin). To illustrate the location, shape and size of FC in S8, an area of the FCE (surrounded by a dotted green line) and the FC borders are highlighted in the right column. From S9 onward, the circumferential MT-orientation no longer exists. Diffuse MT enclose the FC nuclei (dark regions in the centre of the cells) in a basket-like arrangement. **a** During S9–12, the squamous FC covering the NC show a uniform organisation of MT. No preferential MT-orientation referred to the follicle’s axes is discernible in these FC. **b** In the columnar FC, in addition to the basket-like arrangement, a longitudinal MT-orientation develops during S9–12 (parallel green lines at the right margin): In S9, the MT of cFC and mbFC first become oriented along the a-p axis of the follicle. From S10a-12, this pattern spreads out to the pFC. The two right columns show magnifications of boxed areas in the two left columns. For abbreviations, see legend to Fig. 1. Scale bars refer to all pictures in the same column

A detailed analysis of the MT-organisation also revealed characteristic stage-specific patterns during S8–12 (Fig. 5). In S8, similar to bMF, the preferred orientation of MT in the cuboidal FC is perpendicular to the a-p axis of the follicle. From S9 onward, diffuse MT surround the FC nuclei in a basket-like arrangement. In the flattening cFC, a longitudinal orientation of MT along the a-p axis first becomes obvious. During S10a-12, this longitudinal pattern continuously spreads out to mbFC and pFC. During S9–12, the MT of squamous FC covering the NC are organised in typical web-like structures enclosing the nuclei (Fig. 5).

### Bioelectrical patterns correlate with cytoskeletal patterns

**Fig. 6:**
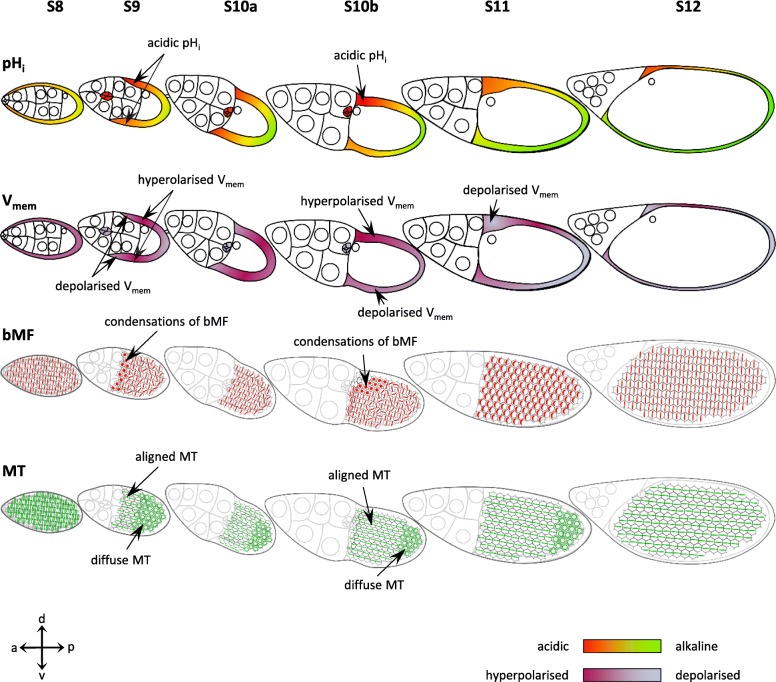
Changes in bioelectrical properties correlate with changes in cytoskeletal patterns in the FCE. Schematic drawings of follicles showing pH_i_ and V_mem_ (according to [16]) and the cytoskeletal organisations (Figs. 4 and 5) in the FCE during S8–12. **pH**_**i:**_ Beginning with S9, an a-p pH_i_-gradient develops with relatively acidic cFC and relatively alkaline pFC. From S10b onward, a d-v gradient establishes with relatively acidic dorsal FC and relatively alkaline ventral FC. **V**_**mem**_: Beginning with S9, an a-p V_mem_-gradient develops with relatively depolarised cFC and pFC, and relatively hyperpolarised mbFC. From S10b onward, a d-v gradient establishes with relatively hyperpolarised dorsal FC and relatively depolarised ventral FC. In late S10b/S11, the dorsal cFC and neighbouring FC become again more depolarised. **bMF**: In S8, the bMF in all FC are aligned in parallel perpendicular to the a-p axis. In S9, the bMF of flattening cFC condense and, in S10a, become aligned in parallel again. In dorsal cFC during S10b, condensation and subsequent disintegration of bMF occur, and this pattern spreads out toward pFC in S11. **MT:** The transversal orientation of MT in S8 changes during later stages: In S9, the MT of cFC become aligned along the a-p axis, whereas the MT of mbFC and pFC are diffusely organised. During S10a-12, the longitudinal orientation of MT spreads out toward pFC. The following correlations become obvious: FC showing condensed bMF (cFC in S9, dorsal cFC and neighbouring FC in late S10b/S11) are relatively acidic and relatively depolarised. Parallel alignment of bMF was observed in relatively alkaline FC, independent of V_mem_ (all FC in S8, mbFC and pFC in S9 and S10a, ventral mbFC and pFC in S10b, all FC in S12). Longitudinal orientation of MT was detected in more acidic FC, independent of V_mem_ (cFC in S9–12, dorsal mbFC in S10a-12), or in more alkaline FC with depolarised V_mem_ (ventral mbFC in S10a-12, pFC in S12)

We have shown previously [15, 16] that, during the course of development, ovarian follicles undergo significant changes in their pH_i_- and V_mem_-patterns caused by varying activities of asymmetrically distributed or activated ion-transport mechanisms. In the present study, we analysed in detail how the cytoskeletal organisation in the FCE alters during vitellogenesis (Figs. 4 and 5). It is obvious that stage-specific changes of pH_i_ and V_mem_ correlate spatially and temporally with structural modifications of bMF and MT (summarised in Fig. 6). These alterations are accompanied by cell migrations, cell rearrangements, or cell-shape changes like, e.g., cell flattening or cell stretching. In S8, the uniformly cuboidal FCE exhibits relatively homogeneous pH_i_- and V_mem_-patterns as well as homogeneous bMF- and MT-patterns [16]. During S9, gradients of pH_i_ and V_mem_ develop with relatively acidic and relatively depolarised cFC [16]. At this stage, the bMF in the flattening cFC lose their circumferential orientation and condense, while the MT change their orientation from d-v to a-p. In S10a, the bMF-bundles of the columnar FCE are aligned in parallel circumferentially again. During further development, d-v gradients of pH_i_ and V_mem_ develop [16]. In S10b, the dorsal FCE is relatively hyperpolarised and relatively acidic compared to the ventral FCE and, as a result, dorsal cFC and neighbouring FC are the most acidic FC. In these, in part, inwardly migrating cells, the bMF condense again. During this process, in late S10b/11, a strong depolarisation of dorsal cFC and neighbouring FC becomes apparent. Unlike the bMF-pattern, the MT-organisation alters gradually along the a-p axis, but not along the d-v axis. In pFC, which are relatively alkaline and depolarised [16], no longitudinal alignment of MT was found.

### Modifying pH_i_ and V_mem_ with inhibitors of ion-transport mechanisms

**Fig. 7:**
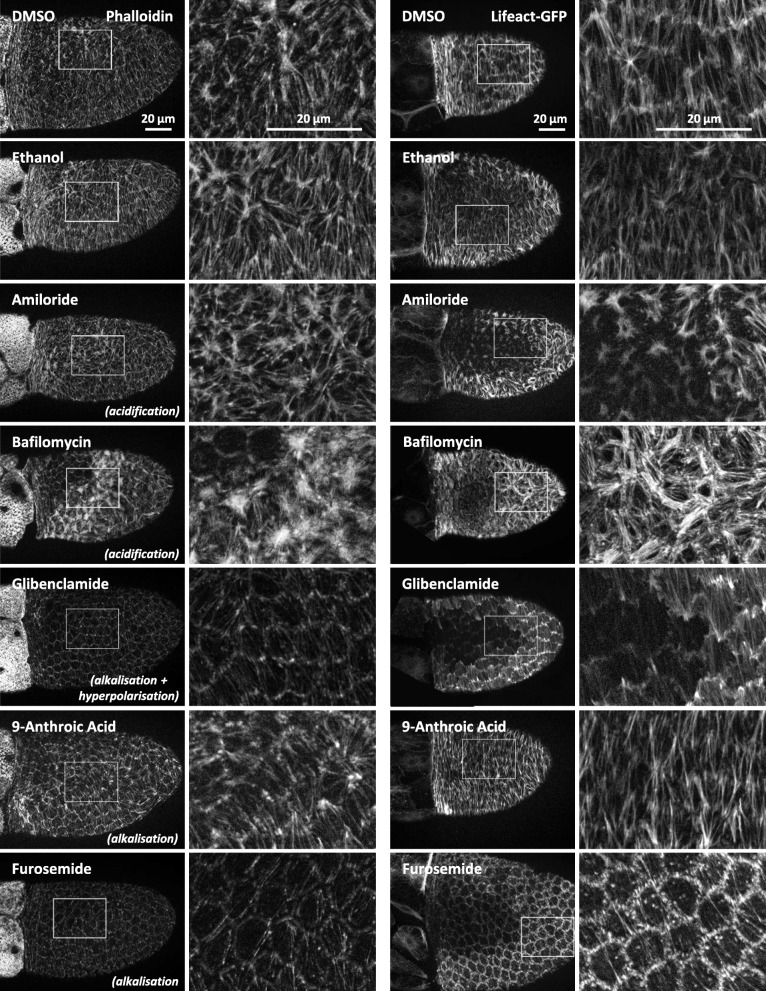
The bMF-organisation is affected by alterations of both pH_i_ and V_mem_. Using inhibitors of ion-transport mechanisms, we modified pH_i_ and/or V_mem_ as well as the bMF-pattern. The results obtained with living Lifeact-GFP follicles (right columns) were similar to those obtained with fixed wild-type follicles using fluorescent phalloidin (left columns). Typical follicles of S10b are shown. For summary, see Fig. 8. Scale bars refer to all pictures in the same column. The inhibitors glibenclamide (ATP-sensitive K^+^-channels) or furosemide (Na^+^/K^+^/2Cl^−^-cotransporters), which both caused strong alkalisation (cf. Figure 2), resulted in parallel alignment of bMF in all FC (control DMSO). Glibenclamide, which led to (moderate) hyperpolarisation, stabilised the bMF-bundles, while furosemide, which had no clear effect on V_mem_, caused partial disintegration of bMF. The inhibitor 9-anthroic acid (Cl^−^-channels), which resulted in slight alkalisation and no clear effect on V_mem_ (cf. Figure 2), also reduced the frequency of bMF-condensations (control ethanol). On the other hand, bafilomycin (V-ATPases) or amiloride (Na^+^/H^+^-exchangers, Na^+^-channels), both acidifying inhibitors with no strong impact on V_mem_ (cf. Figure 2), led to an increasing area of bMF-condensation followed by disintegration of bMF in cFC and dorsal mbFC

**Fig. 8:**
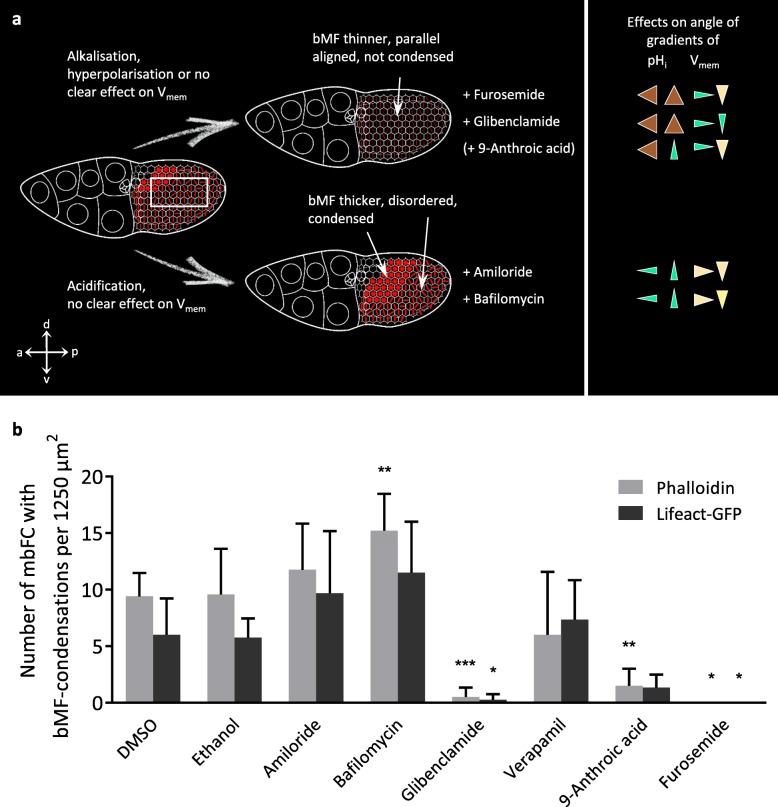
Schematic summary of effects of pH_i_ and/or V_mem_ on bMF, and quantification of bMF-condensations (cf. Figure 7). **a** Alkalisation (together with hyperpolarisation or no clear effect on V_mem_) caused by glibenclamide, furosemide or 9-anthroic acid prevented condensation of bMF (see **b**) and stabilised their parallel alignment, while the bMF-bundles became thinner. This was accompanied by enhanced angles of pH_i_-gradients and reduced angles of V_mem_-gradients (shown on the right, cf. Figure 2). In contrast, acidification (together with no clear effect on V_mem_), caused by amiloride or bafilomycin, led to an increasing area of bMF-condensation (see **b**) in both the a-p and the d-v direction. Moreover, bMF-bundles appeared to be thicker and more disordered. This was accompanied by reduced angles of pH_i_-gradients and no clear effects on angles of V_mem_-gradients (shown on the right, cf. Figure 2). **b** Quantification of bMF-condensations in mbFC (for region of interest, see box marked in **a**) supported the results described above. Mean values, shown with their standard deviation, were compared with the respective controls using an unpaired t-test (3 ≤ *n* ≤ 8; * *p* < 0.05; ** *p* < 0.01; *** *p* < 0.001). Verapamil (slight alkalisation combined with strong hyperpolarisation) resulted either in condensation or in depolymerisation of bMF

**Fig. 9:**
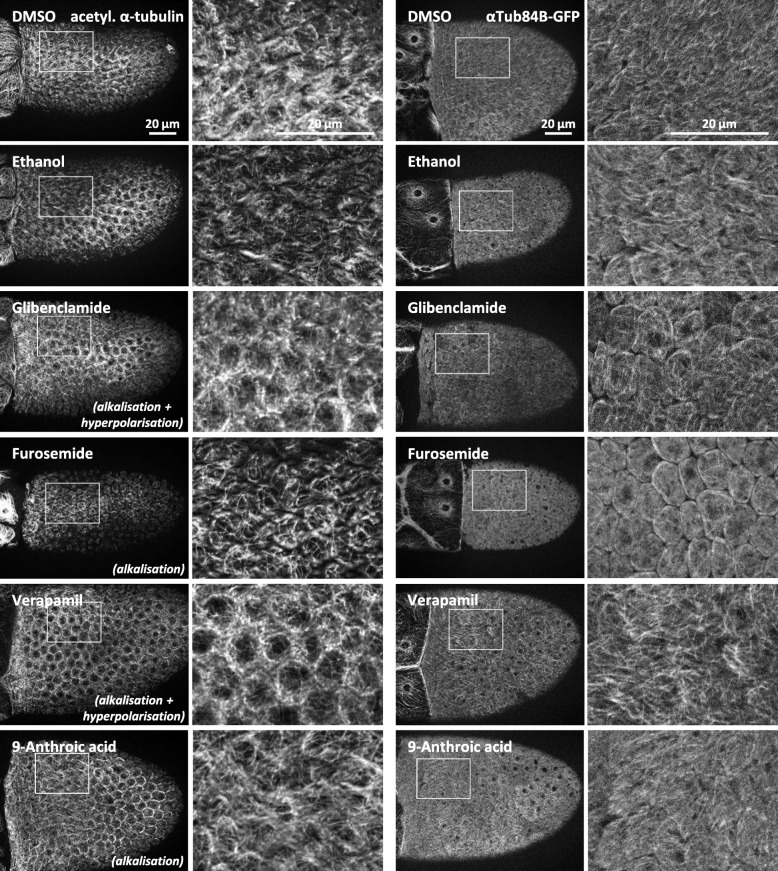
The MT-organisation is affected by alterations of both pH_i_ and V_mem_. Using inhibitors of ion-transport mechanisms, we modified pH_i_ and/or V_mem_ as well as the MT-pattern. The results obtained with living αTub84B-GFP follicles (right columns) were similar to those obtained with fixed wild-type follicles using an antibody against acetylated α-tubulin (left columns). Typical follicles of S10b are shown. For summary, see Fig. 10. Scale bars refer to all pictures in the same column. The inhibitor verapamil (voltage-dependent L-type Ca^2+^-channels), which had the strongest impact on V_mem_ (strong hyperpolarisation, reduction of the angles of the a-p and the d-v gradient, cf. Figure 2) and led to alkalisation, stabilised the longitudinal alignment of MT. In addition, the MT-bundles appeared to be thicker than in the control (ethanol). On the other hand, the MT of follicles treated with either furosemide (Na^+^/K^+^/2Cl^−^-cotransporters; no clear effect on V_mem_; strong alkalisation, cf. Figure 2) or glibenclamide (ATP-sensitive K^+^-channels; moderate hyperpolarisation; strong alkalisation, cf. Figure 2) lost their longitudinal alignment and became partially disintegrated. This coincided with a spherical FC shape, which was especially noticeable in αTub84B-GFP (control DMSO). With 9-anthroic acid (Cl^−^-channels; no clear effect on V_mem_; slight alkalisation, cf. Figure 2), this effect was weaker (control ethanol)

**Fig. 10:**
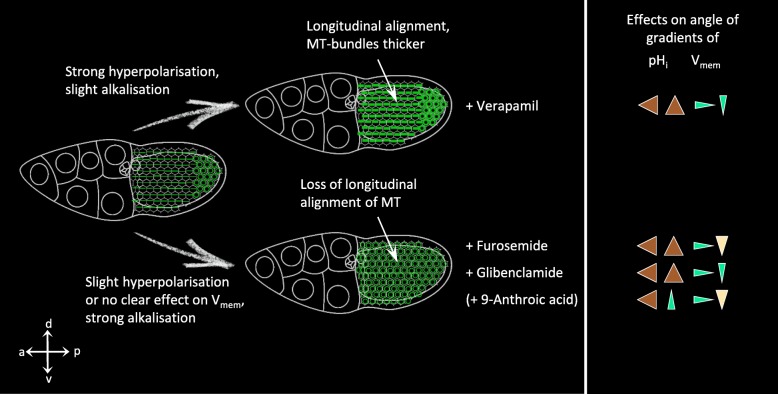
Schematic summary of effects of pH_i_ and/or V_mem_ on MT (cf. Figure 9). Alkalisation (together with slight hyperpolarisation or no clear effect on V_mem_) caused by furosemide, glibenclamide or 9-anthroic acid led to diffuse MT in all FC and to loss of their longitudinal alignment. This was accompanied by enhanced angles of pH_i_-gradients and reduced angles of V_mem_-gradients (shown on the right, cf. Figure 2). Slight alkalisation together with strong hyperpolarisation as well as enhanced angles of pH_i_-gradients and reduced angles of V_mem_-gradients (verapamil; shown on the right, cf. Figure 2) maintained the longitudinal MT-organisation. This was complemented by thickening of the MT-bundles

We used six inhibitors of ion-transport mechanisms, which we have recently shown to affect either pH_i_, V_mem_ or both parameters in the FCE during S10b [16]. We found that each tested inhibitor also exerted influence on the cytoskeletal organisation (Figs. 7, 8, 9 and 10). Certain groups of inhibitors giving rise to similar effects on pH_i_ and/or V_mem_ caused similar changes in the bMF- and/or MT-patterns. Therefore, we conclude that the observed cytoskeletal changes depended on the induced pH_i_- and/or V_mem_-changes, and not on effects of the involved ions.

As described in detail previously ([16], summarised in Fig. 2), alkalisation was caused by furosemide, glibenclamide, 9-anthroic acid or verapamil. Furosemide and glibenclamide resulted in the strongest overall increase of pH_i_ and also in enhanced angles of the a-p and the d-v gradient. 9-Anthroic acid led to an enhanced angle of the a-p gradient, but to a reduced angle of the d-v gradient. Amiloride or bafilomycin resulted in acidification and in reduction of the angles of the a-p and the d-v gradient. V_mem_ was influenced to the greatest extent by verapamil (strong hyperpolarisation), followed by glibenclamide (hyperpolarisation), and both inhibitors reduced the angles of the a-p and the d-v gradient. Furosemide, 9-anthroic acid, amiloride and bafilomycin, respectively, had no consistent effects on V_mem_ and on both gradients.

### Changes in pH_i_ and V_mem_ affect the organisation of basal microfilaments

Inhibition experiments were performed using S10b-follicles of the wild-type as well as of the transgenic strain Lifeact-GFP. The bMF-patterns in the FCE of both strains were very similar (Figs. 7 and 8), only a slight difference in the thickness of bMF-bundles was obvious: The bMF-bundles of fixed phalloidin-stained wild-type follicles were thinner than those of living Lifeact-GFP follicles. Furthermore, Lifeact-GFP follicles often showed a weakly fluorescent area in the FCE that seemed to result from squeezing during microscopic observation.

Despite of some variation, the effects of inhibitors on the bMF-patterns were also similar in both strains (Figs. 7 and 8). Strong alkalisation, either without a distinct effect on V_mem_ (furosemide) or combined with hyperpolarisation (glibenclamide), retained a highly polarised bMF-pattern consisting of parallel aligned, but thinner bMF-bundles, whereas condensations of bMF, as in the controls, were rarely observed (Fig. 8a,b). The bMF-bundles of furosemide-treated follicles appeared to be even thinner and partially disintegrated compared to those of glibenclamide-treated follicles. Presumably, this difference depends on the fact that furosemide showed no clear influence on V_mem_. Furosemide and glibenclamide both led to alkalisation in all FC, but especially in pFC and ventral FC, thus enhancing the angles of the a-p and the d-v pH_i_-gradient (cf. Figure 2). Both V_mem_-gradients were either maintained or reduced resulting in a larger area of relatively hyperpolarised FC.

Slight alkalisation together with no clear effect on V_mem_ (9-anthroic acid) reduced the frequency of bMF-condensations (Fig. 8a,b). This treatment led to an enhanced angle of the a-p pH_i_-gradient but to a reduced angle of the d-v pH_i_-gradient, since the ventral cFC became less alkalised (cf. Figure 2). Slight alkalisation combined with strong hyperpolarisation (verapamil) resulted either in depolymerisation or in condensation of bMF throughout the entire columnar FCE (Fig. 8b). This seems to be due to the fact that the angles of both the a-p and the d-v V_mem_-gradient were reduced, which led to more homogeneous electrochemical properties throughout the FCE (cf. Figure 2).

Acidification combined with an unchanged V_mem_ (amiloride, bafilomycin) led to an increase in bMF condensation and disintegration. The angles of the a-p and the d-v pH_i_-gradient of both amiloride- and bafilomycin-treated follicles were reduced (cf. Figure 2), and the relatively acidic area of the FCE showing condensed bMF was enlarged (Fig. 8a,b).

Taken together, we found that alkalisation prevented condensation of bMF and stabilised their parallel alignment, while the bMF-bundles became thinner. In contrast, acidification led to increasing condensations of bMF in both the a-p and the d-v direction, while the bMF-bundles became thicker and more disordered. When strong alkalisation was combined with hyperpolarisation, disintegration of bMF was absent. Thus, hyperpolarisation had a stabilising effect on bMF (Figs. 7 and 8).

### Changes in pH_i_ and V_mem_ affect the organisation of microtubules

Inhibition experiments were performed using S10b-follciles of the wild-type as well as of the transgenic strain αTub84B-GFP. While, in living αTub84B-GFP follicles, the α-subunits of all MT in the FCE were labelled, only a subset of MT was stained in fixed wild-type follicles treated with an antibody against acetylated α-tubulin. Thus, in αTub84B-GFP, a denser network of MT-bundles was revealed and the overall longitudinal alignment of MT became more evident (Figs. 9 and 10).

Alkalisation, caused by furosemide, glibenclamide or 9-anthroic acid, resulted either in reduction (glibenclamide, 9-anthoric acid) or in loss (furosemide) of the longitudinal orientation of MT as well as in their partial disintegration. In furosemide-treated follicles (strong alkalisation, no clear effect on V_mem_), disintegration of MT was most prominent compared to follicles treated with glibenclamide (strong alkalisation, slight hyperpolarisation) or with 9-anthoric acid (slight alkalisation, no clear effect on V_mem_). Strong alkalisation combined with no clear effect on V_mem_ (furosemide) resulted in spherical FC, presumably due to weakend cell-cell contacts, which was particularly visible in αTub84B-GFP. This phenomenon was less pronounced with glibenclamide, presumably due to a stabilising effect of hyperpolarisation. Slight alkalisation combined with strong hyperpolarisation (verapamil) preserved the longitudinal orientation, while the MT-bundles appeared to be thicker (Figs. 9 and 10).

In addition, furosemide, glibenclamide or 9-anthroic acid led to an enhanced angle of the a-p pH_i_-gradient and to a reduced angle of the a-p V_mem_-gradient (cf. Figure 2). This means that the cFC became more alkaline compared to the mbFC, while the area of relatively hyperpolarised FC became enlarged. The altered pH_i_-gradient resulted in loss of the longitudinal MT-alignment in the mbFC and cFC. Verapamil also led to an enhanced angle of the a-p pH_i_-gradient and to a reduced angle of the a-p V_mem_-gradient (cf. Figure 2). But the effect of verapamil on pH_i_ in general as well as on its gradients was small, so that it had no impact on MT-organisation. In addition, the strong hyperpolarising effect of verapamil and the reduced V_mem_-gradients both preserved the longitudinal alignment of MT (Figs. 9 and 10).

Acidification in the whole FCE as well as reduced angles of both pH_i_-gradients combined with no clear effects on V_mem_ caused by amiloride and bafilomycin (cf. Figure 2) did not alter the MT-organisation (not shown).

Therefore, we conclude that hyperpolarisation as well as acidification exerted stabilising effects on the longitudinal orientation of MT-bundles. Strong alkalisation resulted in loss of this MT-arrangement and in partial disintegration of MT. These effects were reduced when alkalisation was combined with hyperpolarisation, which preserved the longitudinal orientation of MT-bundles (Figs. 9 and 10).

## Discussion

**Fig. 11:**
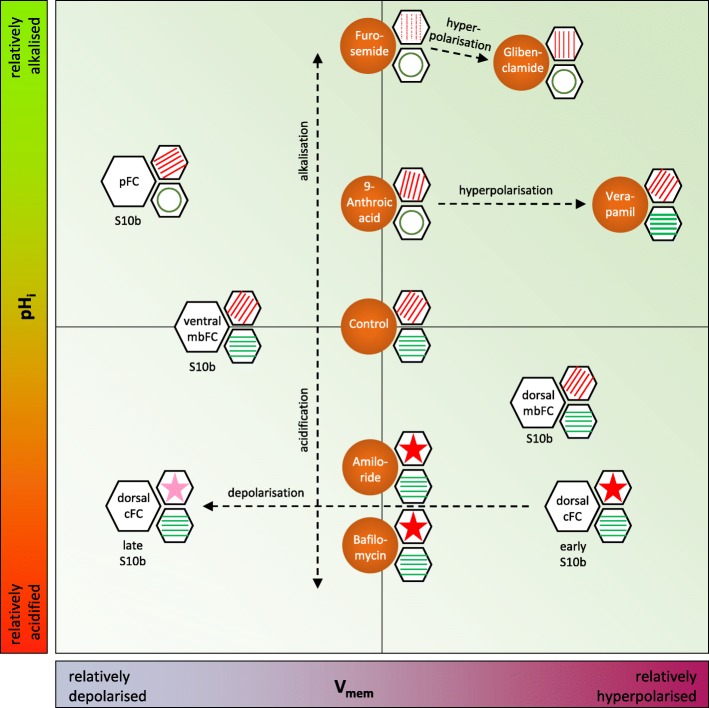
Schematic representation of correlations between pH_i_, V_mem_, bMF and MT. The different FC types in S10b (cf. Figure 1) are symbolised by white hexagons, and the applied inhibitors by orange circles. The organisation of bMF in FC is symbolised by red lines (parallel orientation: d-v or oblique; dotted: disintegration) or by red asterisks in hexagons (dark red: condensation; light red: condensation and disintegration). The organisation of MT in FC is symbolised by green lines (parallel orientation: a-p; thick line: thickening) or by green circles in hexagons (loss of parallel orientation and partial disintegration). Dotted arrows indicate changes of pH_i_ (relative acidification or alkalisation; left margin) and changes of V_mem_ (relative hyperpolarisation or depolarisation; bottom margin) relative to the control state (centre). The correlations between pH_i_, V_mem_, bMF and MT observed in different FC types during S10b are in line with the correlations resulting from the inhibition of Na^+^/H^+^-exchangers and Na^+^-channels (amiloride), V-ATPases (bafilomycin), ATP-sensitive K^+^-channels (glibenclamide), voltage-dependent L-type Ca^2+^-channels (verapamil), Cl^−^-channels (9-anthroic acid) or Na^+^/K^+^/2Cl^−^-cotransporters (furosemide). While alkalisation and/or hyperpolarisation stabilises the parallel transversal alignment of bMF, acidification leads to increasing disorder and to condensations of bMF. On the other hand, acidification as well as hyperpolarisation stabilises the longitudinal orientation of MT, whereas alkalisation leads to loss of this arrangement and to partial disintegration

Considering the results of our analysis of stage-specific patterns as well as of inhibition experiments, correlations between pH_i_- and V_mem_-changes and changes of the cytoskeletal organisation become obvious. Alkalisation supports the parallel alignment of bMF-bundles and prevents the longitudinal orientation of MT, whereas acidification results in increasing condensation and subsequent disintegration of bMF while supporting the longitudinal alignment of MT. Depending on pH_i_, hyperpolarisation has stabilising effects on bMF- or on MT-bundles, whereas depolarisation correlates with bMF-disintegration or with reduced longitudinal MT-orientation (summarised in Fig. 11). Obviously, bMF and MT are not disintegrated under the same electrochemical conditions. It seems as if bMF are predominantly stabilising the transversal axis while MT are stabilising the longitudinal axis of the follicle.

Our observations are in line with previous findings in various systems. Besides a pH-dependence of actin self-assembly [23], it has been shown that contractility of the actomyosin cytoskeleton [24] as well as the activities of cross-linking proteins, like cortexillin, filamin and fascin, are regulated by pH [25, 26]. The interplay between actin, myosin and cross-linking proteins is highly complex, and the pH-dependencies of these proteins are diverse. Accordingly, specific changes of the cytoskeletal organisation in consequence of pH_i_-modifications relate to the respective cytoplasmic mixture of actin-binding proteins [25]. Also indirect effects, e.g. changes in cell volume or cell tension, could be envolved.

Condensation of bMF was mainly observed in FC that undergo significant morphological changes, like flattening and stretching. This applies for the cFC in S9 and for the dorsal cFC and neighbouring FC, the prospective floor and roof cells of respiratory appendages [54, 55], in S10b. Since cell-shape changes require a reorganisation of the cytoskeleton, we assume that condensation and subsequent disintegration of bMF are distinct steps of cytoskeletal restructuring processes. Reorganisation of the bMF-network is usually correlated with lower pH_i_. It has been shown in vitro that acidification leads to increased bundling of MF and to subsequent contraction of the actomyosin network, while alkalisation maintains the MF-organisation by stabilising cross-linking [24]. The described condensation of bMF in the relatively acidic cFC in S9 and dorsal cFC in S10b, as well as in the mbFC after treatment with amiloride or bafilomycin, seems to be based on a comparable mechanism. On the other hand, the parallel alignment of bMF in relatively alkaline FC, for example after application of glibenclamide or furosemide, indicates stable cross-linking between bMF-bundles. This interpretation is further supported by the observation that actin self-assembly is accelerated at lower pH_i_ [23], since restructuring of the MF-network depends on rapid self-assembly.

The MT-organisation has been shown to be directly influenced by pH_i_-changes: Acidification results in polymerisation of tubulin while alkalisation results in depolymerisation [25, 29, 30]. Loss of the longitudinal alignment of MT observed in the relatively alkaline pFC and in all FC after the application of alkalising inhibitors (furosemide, glibenclamide or 9-anthroic acid) is likely to be based on MT-depolymerisation (Fig. 11).

In addition to being regulated by pH_i_-changes, both MF and MT are known to be affected by V_mem_-changes. In cultured bovine corneal endothelial cells, it has been demonstrated that depolarisation of V_mem_ leads to reorganisation and to decreasing densities of the MF- and MT-networks [31]. Depolarisation is usually correlated with MF-network restructuring processes, whereas hyperpolarisation is correlated with stabilisation of the MF-organisation [27, 28]. These findings are in line with our own observations (Fig. 11). Moreover, since MF as well as MT are charged and polar polymers that can act as electrical conductors, both cytoskeletal networks are highly sensitive to electrical fields [32, 33, 56].

## Conclusion

Correlations between stage-specific bioelectrical properties and cytoskeletal patterns observed in the FCE of *Drosophila* were confirmed by the application of inhibitors of several ion-transport mechanisms. We conclude that the changes of pH_i_- and V_mem_-gradients induced by inhibitors simulate electrochemical changes that occur naturally, resulting in the cytoskeletal changes observed during differentiation of the FCE. Our results support the hypothesis that electrochemical signals play important roles in the regulation of cell and tissue architecture by organising elements of the MF- and MT-cytoskeleton. It remains to be shown which specific elements are affected by these signals.

## Methods

### Preparation of follicles

*Drosophila melanogaster* were reared at 20–23 °C on standard medium with additional fresh yeast. 2–3 days old females were killed by crushing the head and thorax with tweezers without anaesthesia. The ovaries were dissected and single follicles of vitellogenic stages (S8–12) were isolated (see Fig. 1). The preparations were carried out in R-14 medium [57] which is best suited for in-vitro culture of *Drosophila* follicles [58].

In addition to wild-type (Oregon R), we used the Gal4/UAS system for the follicle-cell specific expression (Tj-Gal4; gift of S. Roth, Köln, Germany) of GFP-actin (UAS-Lifeact-GFP; Bloomington Stock Center, USA) and GFP-α-tubulin (UAS-αTub84B; Bloomington), respectively.

### Labelling of microfilaments

Follicles were fixed for 20 min in microfilament-stabilising buffer (MF-buffer) containing 4% formaldehyde according to [47], washed in phosphate-buffered saline (PBS) and stained for 20 min with 0.25 μg/ml phalloidin-FluoProbes 550A (Interchim, France; dissolved in dimethyl sulfoxide, DMSO) which specifically binds to F-actin.

### Indirect immunofluorescence labelling of microtubules

Follicles were fixed for 20 min in MF-buffer, washed in PBS and blocked for 1 h with 2% bovine serum albumin (BSA)/0.1% Triton X-100 in PBS. Thereafter, the follicles were incubated for 1 h at 20 °C or overnight at 4 °C in PBS containing 1% BSA/0.1% Triton X-100 and a monoclonal antibody against acetylated α-tubulin (6-11B-1; Santa Cruz Biotechnology, USA; diluted 1:100). After washing, the follicles were treated with goat-anti-mouse biotin (Dianova, Germany; diluted 1:200) for 1 h in PBS containing 1% BSA/0.1% Triton X-100. Washing was repeated before TexasRed-conjugated streptavidin (Dianova; diluted 1:1000) was added for 30 min in PBS containing 1% BSA/0.1% Triton X-100.

### Fluorescence microscopy and optical sectioning

Fixed follicles were imaged in Fluoromount G (Interchim), and living follicles in R-14 medium, respectively, using a Zeiss AxioImager.M2 structured-illumination microscope, equipped with a Zeiss ApoTome and a Zeiss AxioCamMRm camera using a × 40/1.3oil objective and the appropriate filter sets. To investigate either basal microfilament (bMF) or microtubule (MT) patterns, tangential optical sections of follicles were analysed (Fig. 3).

### Inhibition of ion-transport mechanisms

All S10b-follicles of a single fly (approximately 10–20 follicles) were divided into a control group and an experimental group. Inhibition was performed for 20 min in R-14 medium containing the respective inhibitor according to [16]. The following inhibitors of ion-transport mechanisms were used: Na^+^/H^+^-exchangers (NHE) and Na^+^-channels were blocked with amiloride (Sigma-Aldrich, Germany; 10 μM; dissolved in DMSO), V-ATPases with bafilomycin A1 (Sigma-Aldrich; 160 nM; dissolved in DMSO), ATP-sensitive K^+^-channels with glibenclamide (Biomol, Germany; 100 μM; dissolved in DMSO), voltage-dependent L-type Ca^2+^-channels with verapamil-HCl (Sigma-Aldrich; 50 μM; dissolved in ethanol), Cl^−^-channels with 9-anthroic acid (Sigma-Aldrich; 100 μM; dissolved in ethanol), and Na^+^/K^+^/2Cl^−^-cotransporters with furosemide (Sigma-Aldrich; 1 mM; dissolved in DMSO). Control experiments were performed in R-14 medium containing 0.1–1% v/v ethanol or DMSO without the respective inhibitor. After treatment, wild-type follicles were fixed and stained before analysis while GFP-follicles were directly analysed as described above. Each experiment was performed at least three times.

## Data Availability

The datasets used during the current study are available from the corresponding author on reasonable request.

## Abbreviations

a-p: Antero-posterior
bMF: Basal microfilaments
cFC: Centripetal follicle cells
DIC: Differential interference contrast
DMSO: Dimethyl sulfoxide
d-v: Dorso-ventral
FC: Follicle cell
FCE: Follicle-cell epithelium
mbFC: Mainbody follicle cells
MF: Microfilaments
MF-buffer: Microfilament-stabilising buffer
MT: Microtubules
NC: Nurse cells
NHE: Na^+^/H^+^-exchangers
Oo: Oocyte
PBS: Phosphate buffered saline
pFC: Posterior follicle cells
pH_i_: Intracellular pH
S: Stage
V_mem_: Membrane potential
